# 

**Published:** 1891-03-28

**Authors:** 


PRICE ONE PENNY.

No. 235, Vol. IX.—March 28th, 1891.

[Registered at the General Post Office as a Newspaper for transmission in the United Kingdom and Abroad.]

WITH EXTRA NURSING SUPPLEM

Per Annum, 6s. 6d.; post free.

Monthly Parts, 6d.; post free 8d.

Ditto per Annum, 8s., post free.

### THE HOSPITAL

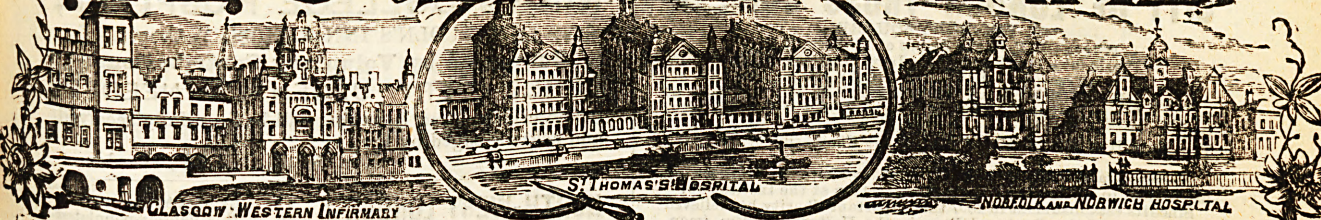

A WEEKLY JOURNAL OF

### Science, Medicine, Nursing and Philanthropy.

### SATURDAY, MARCH 28th, 1891.

**The Midwives' Bill: A Select Committee** - - - 367
**Passing Topics.**—The Jubilee Institute for Nurses - 368
**The Doctor's Armchair.**—A New Interpreter of Faith - 369
**The History and Capabilities of Herbal Simples.** By W. T. Fernie, M.D. XXIX.—Dill - - - - 370
**Books Received** - - - - - - 370
**Everybody's Page** - - - - - - 371
**Editor's Letter Box.**—Medical Women and the Royal Free Hospital—Body and Mind at Cambridge - - - 371
**Notes and News** - - - - - - 372
**Scraps and Gleanings** - - - - - 373
**The Lords' Committee** - - - - - 378
**Annotations.**—The Royal Free Hospital and Medical Women —A Crisis in Child Labour—"Chronic" Students - - 374
**The Practitioner's Mirror and Retrospect:**—
Medicine.—Erythro-Melalgia—Diseases of the Intracranial Vessels in Children, 375; Herpes - - - - - - 376
Surgery.—The Pathological and Surgical Treatment of Suppurative Tonsilitis - - - - - - 377
Therapeutics.—Dithiosalicylate of Soda - - - - 377
**The Registration of Midwives** - - - - - 378
**The Student of Medicine.**—Notes from the Schools—Students' Medical Societies—Appointments—Vacancies—Pass Lists—Athletics.

### EXTRA SUPPLEMENT—THE NURSING MIRROR.

**En Passant.**—Short Items—The Jubilee Institute—Rash Accusations—Portsmouth News—Starting a New Society—Infected Clothing by Parcels Post - - cxliii

**Lectures on Surgical Ward Work and Nursing.** By Alexander Miles, M.B.Edin., C.M., F.R.C.S.E. Lecture XVIII.—After Operation - - - cxliv

**The Eastern Hospital Scandal** - - - - cxlv

**Death in Our Ranks** - - - - - - cxlv

**For Reading to the Sick.**—The Invitation - - - - cxlv

**Appointment** - - - - - - - - cxlv

**Presentation by the First Thousand** - - - - cxlvi

**Notes from Australia** - - - - - - cxlvii

**Off Duty.**—Through the Hoop - - - - - cxlviii

**Princess Christian's Daughter** - - - - - cxlviii

**Amusements and Relaxation** - - - - - cxlviii

